# Transcription Factor Reprogramming in the Inner Ear: Turning on Cell Fate Switches to Regenerate Sensory Hair Cells

**DOI:** 10.3389/fncel.2021.660748

**Published:** 2021-03-29

**Authors:** Amrita A. Iyer, Andrew K. Groves

**Affiliations:** ^1^Department of Molecular and Human Genetics, Baylor College of Medicine, Houston, TX, United States; ^2^Program in Genetics & Genomics, Houston, TX, United States; ^3^Department of Neuroscience, Baylor College of Medicine, Houston, TX, United States

**Keywords:** transcription factors, reprogramming, pioneer factor, inner ear, hair cell, regeneration

## Abstract

Non-mammalian vertebrates can restore their auditory and vestibular hair cells naturally by triggering the regeneration of adjacent supporting cells. The transcription factor ATOH1 is a key regulator of hair cell development and regeneration in the inner ear. Following the death of hair cells, supporting cells upregulate ATOH1 and give rise to new hair cells. However, in the mature mammalian cochlea, such natural regeneration of hair cells is largely absent. Transcription factor reprogramming has been used in many tissues to convert one cell type into another, with the long-term hope of achieving tissue regeneration. Reprogramming transcription factors work by altering the transcriptomic and epigenetic landscapes in a target cell, resulting in a fate change to the desired cell type. Several studies have shown that ATOH1 is capable of reprogramming cochlear non-sensory tissue into cells resembling hair cells in young animals. However, the reprogramming ability of ATOH1 is lost with age, implying that the potency of individual hair cell-specific transcription factors may be reduced or lost over time by mechanisms that are still not clear. To circumvent this, combinations of key hair cell transcription factors have been used to promote hair cell regeneration in older animals. In this review, we summarize recent findings that have identified and studied these reprogramming factor combinations for hair cell regeneration. Finally, we discuss the important questions that emerge from these findings, particularly the feasibility of therapeutic strategies using reprogramming factors to restore human hearing in the future.

## Introduction

Hearing loss is a globally prevalent disorder characterized by one or a combination of loss of inner ear hair cells, malfunction, or degeneration of components critical to hearing such as the stria vascularis, or loss of spiral ganglion neurons or their synaptic connections with hair cells. In practice, assistive devices such as hearing aids, cochlear implants, and auditory brainstem implants are the only current options available to help manage hearing loss, but these cannot fully restore hearing. Regeneration of inner ear hair cells by supporting cells or other non-sensory cells has been an attractive possibility for hearing restoration since its discovery as a naturally occurring phenomenon in non-mammalian vertebrates (Corwin and Cotanche, 1988; Ryals and Rubel, 1988; Cotanche, 1999). Supporting cells can either directly transdifferentiate or re-enter the cell cycle and divide to yield new hair cells. Since then, efforts to translate this phenomenon to mammals have gained traction, intending to treat human hearing loss.

One of several interventions explored is the ectopic expression of hair cell-specific transcription factors such as ATOH1 to reprogram non-sensory inner ear cells into hair cells. Studies over the past 20 years have shown that ATOH1 successfully reprograms non-sensory cells adjacent to the organ of Corti to form hair cells in the neonatal mouse cochlea, and a small number of studies have reported a similar result in older animals, although at far lower efficiency (Kelly et al., 2012; Lee S. et al., 2020). This age-dependent decline in the reprogramming ability of ATOH1 led to a search for additional transcription factors to reprogram older cochlear cells into hair cells. In this review, we focus on the potential for transcription factor reprogramming in the adult inner ear. We also discuss the potential of different cochlear cell types to serve as reprogramming reservoirs within the mammalian inner ear.

## Cellular Reprogramming: Towards A Pluripotent Cell Fate

What we now refer to as cellular reprogramming was first demonstrated by John Gurdon through the process of somatic cell nuclear transfer in frogs in the 1950s. His experiments showed that nuclei from tadpole intestinal epithelial cells led to the development of a normal tadpole when transferred to an enucleated egg (Gurdon, 1962). Following this, virus-mediated cell fusion experiments coupled with microsurgical removal of zygotic pronuclei were carried out in mice. When donor nuclei were introduced into enucleated mouse zygotes, the resulting embryos developed comparably normally to those derived from unmanipulated zygotes (McGrath and Solter, 1983). Cloning experiments in sheep demonstrated that donor nuclei from fetal and adult mammary gland cells could produce healthy embryos when transferred into unfertilized eggs (Willadsen, 1986; Wilmut et al., 1997). Though the field lacked a detailed molecular and genetic understanding of reprogramming at that point, these experiments provided definitive evidence for the presence of factors in the egg cytoplasm that were capable of restoring the chromatin of a differentiated cell to something resembling its original pluripotent state (DiBerardino et al., 1984).

Although the experiments described above relied on cytoplasmic factors to elicit reprogramming, the known ability of transcription factors to drive cell fate conversion led to the search for transcription factors that could reprogram differentiated cells back to a pluripotent state. A unique cocktail of transcription factors in pluripotent embryonic stem cells—OCT4, SOX2, KLF4, and C-MYC (designated the OSKM/Yamanaka factors after the laboratory that first identified them)—was demonstrated to reprogram mouse embryonic and adult fibroblasts into a “pre-differentiated” or pluripotent stem cell state (Takahashi and Yamanaka, 2006). The cells that were induced to become pluripotent after reprogramming became known as induced pluripotent stem cells (iPSCs). Cultured iPSCs expressed specific pluripotency markers and possessed embryonic stem cell-like morphology and growth characteristics. When transplanted into immunocompromised nude mice these cells gave rise to cell types from all three germ layers, confirming their pluripotent properties (Takahashi and Yamanaka, 2006). Human iPSCs were generated using the same four OSKM factors by Yamanaka’s group the following year (Takahashi et al., 2007).

Another transcription factor combination comprising SOX2, OCT4, NANOG, and LIN28 was discovered to yield “fate irreversible” pluripotent stem cells (in previous cases, most of the iPSCs reverted to their original fate after 2–3 generations) from human dermal fibroblast cells (Yu et al., 2007; Tanabe et al., 2013). Furthermore, it was shown that NKX-3, a transiently expressed homeobox transcription factor endogenously activated OCT4 and was essential for iPSC reprogramming of mouse and human cells (Mai et al., 2018). The addition of small molecules like Valproic acid (VPA), a histone deacetylase (HDAC) inhibitor, together with OSKM factors improved reprogramming efficiency in mouse fibroblasts by 100-fold (Huangfu et al., 2008). The same study showed that VPA was also successful as a replacement for *c-Myc*, an oncogene whose overexpression in iPSCs was a cause of concern due to its potential tumorigenicity. It was then established that a set of seven small-molecule compounds, namely Valproic acid (VPA, an HDAC inhibitor), FSK (Forskolin, an adenylyl cyclase activator), CHIR (Aminopyramidine derivative, a GSK-3 beta inhibitor), 616452 (a TGF-beta receptor inhibitor), Tranyl (a histone demethylation inhibitor), DZNep (adenosine analog, an EZH2 inhibitor) and TTNPB (Retinoic acid analog, a retinoic acid pathway activator) could replace all four transcription factors to successfully reprogram mouse somatic cells into pluripotent stem cells (Hou et al., 2013).

Mechanistic studies in mouse fibroblasts showed that the induction of pluripotency begins with the repression of fibroblast-specific marker genes, followed by the endogenous expression of the transcription factor genes, *Oct4*, *Sox2*, and *Klf4* (OKS) that are sufficient for a self-sustaining pluripotency state, along with the upregulation of telomerase. Exogenous expression of the OKS factors alone was found to enable multiple somatic cell types to reprogram to an iPSC fate (Maherali et al., 2007; Brambrink et al., 2008; Stadtfeld et al., 2008). OCT4 and SOX2 are known to interact with each other cooperatively to activate OCT/SOX-specific enhancers in genes like *Fbx15* and *Nanog* to maintain pluripotency in mouse embryonic stem cells (Tokuzawa et al., 2003; Masui et al., 2007). Further, it was shown experimentally that NANOG recruits RNA polymerase II and promotes the expression of *Esrrb* that is critical for pluripotency fate in multiple cell phases (pre iPSC, partially/incompletely reprogrammed iPSCs, and developing embryonic stem cells) (Festuccia et al., 2012). Interactome studies showed that almost all pluripotency genes lie within the gene regulatory networks of OCT4, SOX2, and NANOG (Wang et al., 2006). The reprogramming mechanisms of C-MYC include the recruitment of several chromatin remodelers (p400, Ini1, Tip48/49), ubiquitin ligases (Fbw7, Skp2), and histone acetyltransferases (Tip60, p300, GCN5; Adhikary and Eilers, 2005). Within iPSCs, C-MYC predominantly maintains lineage-specific transcription factor genes in a bivalent state (in conjunction with H3K27 methylation marks and the SuZ12 subunit of the PcG repressor complex), altering H3K27 and H3K4 methylation status of target gene promoters for their repression or expression, respectively (Mikkelsen et al., 2007).

iPSC reprogramming is known to suffer from unpredictable and low efficiencies, resulting in heterogeneous populations of iPSCs (Stadtfeld and Hochedlinger, 2010). In human somatic cells, combined single-cell analysis of transcription and chromatin accessibility during reprogramming showed that a switch from gene regulatory networks controlled by FOSL1 to networks regulated by TEAD4 can drive cells towards a pluripotent cell state (Xing et al., 2020). The application of single-cell RNA sequencing and ATAC sequencing to study these reprogrammed heterogenous populations continues to advance our understanding of reprogramming efficiency-associated roadblocks.

The *in vivo* introduction of pluripotency genes adds challenges associated with the negative effects of their genomic integration and continued overexpression. Several pluripotency genes especially *Oct-4*, *Nanog*, *Gdf3*, and *Stella* are known to be expressed in germline tumors (Clark et al., 2004). OCT4 inhibits cellular differentiation resulting in dysplasia of epithelial tissue which supports the notion that a progenitor phase precedes tumorigenesis in adult tissue (Hochedlinger et al., 2005). When applying combinations of reprogramming factors in any tissue, the reprogrammed cell transitions through multiple potential progenitor phases which could lead to malignancy. Recent studies have employed a more transient overexpression model to address tumorigenicity issues. For example, mouse skeletal muscle was regenerated after injury with no tumorigenicity when OSKM factors were transiently overexpressed using plasmids (De Lázaro et al., 2019). Another study showed that transient overexpression of the OSKMLN reprogramming factors through mRNA cocktails improved the progression of aging in progeroid mice (Sarkar et al., 2020). Chromatin remodeling-based modifications for tissue reprogramming through CRISPR/Cas9 targeting have been reviewed recently (Martinez-Redondo and Izpisua Belmonte, 2020). In particular, CRISPR-based strategies devised to have no detectable off-target effects have been proposed for long-term applications (Akcakaya et al., 2018). Incorporating techniques that enhance safety and retain the efficacy of reprogramming is an important consideration when aiming to ultimately treat human disease.

DNA methylation is a heritable epigenetic modifier that controls cell fates. It is promoted by DNA methyltransferases (DNMT1, DNMT3a, DNMT3b) which catalyze the addition of a methyl group to the cytosine residue at specific DNA loci (Hon et al., 2009). DNA methylation at the promoter and enhancer regions of genes prevents the binding of transcription factors, resulting in gene repression (Xie et al., 2013). During the process of differentiation, promoter and enhancer regions of pluripotency genes are hypermethylated as they are down-regulated, allowing for the expression of differentiation genes and the adoption of unique cell fates. Additionally, differential methylation of lineage-specifying gene enhancers results in the production of functionally diverse cell types within the same tissue. For example, a study of adult skin and hematopoietic stem cell differentiation revealed locus-specific methylation changes in different cell types, often associated with the repression in a particular cell type of transcription factors specific for other cell types in that lineage (Bock et al., 2012). Analysis of DNA methylation during reprogramming can be used to identify the fidelity of the reprogramming mechanism—for example, a genome-wide DNA methylation analysis showed that the promoters of *Oct4*, *Nanog*, and *Dnmt3b* were unmethylated in ES cells but partially methylated in iPS cells, providing markers to differentiate between them (Deng et al., 2009). Another study compared the regions of DNA hypo- and hypermethylation between iPSCs and parental fibroblasts to find that complete reprogramming requires extensive DNA methylation alterations (Doi et al., 2009). iPSCs were also found to possess residual DNA methylation marks from the parental cell type with a tendency to re-differentiate (Kim et al., 2010; Polo et al., 2010). Incompletely reprogrammed iPSCs were unable to reactivate pluripotency genes due to persistent hypermethylation of pluripotency gene promoters and incomplete repression of cell type-specific transcription factors (Mikkelsen et al., 2008). Studies of methylation during reprogramming have also been helped by recent advances in sequencing platforms that have enabled the study of cell methylomes at single-nucleotide resolution employing MethylCseq (Lister and Ecker, 2009).

## Direct Cellular Reprogramming: Towards A Specific Cell Fate

Transcription factors are capable of reprogramming one differentiated cell type into another directly without the need to actively induce an intermediate pluripotent stem cell fate. This process is termed direct cellular reprogramming or direct transdifferentiation. Initial cell fusion experiments between human amniocytes and mouse muscle cells showed activation of a muscle cell-specific genetic program in the resultant heterokaryons (Blau et al., 1983). In mice, ectopic expression of *MyoD* in fibroblasts successfully transdifferentiated them into myoblasts (Davis et al., 1987). Its identification came about through a subtraction-hybridization method for cDNAs of genes differentially expressed in myoblasts and not in the mesodermal stem cell line C3H10T1/2. When *MyoD* cDNA was ectopically expressed in 10T1/2 cells, it resulted in stable clones of myogenic cells that were competent enough to undergo further myogenesis. These early results showed the feasibility of direct reprogramming, and we describe recent examples of this strategy in the following sections.

### Recent Attempts at Direct Cellular Reprogramming in the Nervous System, Pancreas, and Heart

Initial attempts to promote direct transdifferentiation of one cell type to another through transcription factor reprogramming used a strategy similar to that used for identifying the OSKM factors—the screening of an initial pool of transcription factors to identify combinations that could promote conversion. In these direct reprogramming efforts, the starting pool of transcription factors was selected based on their known roles in the differentiation of the desired cell type. For example, a transcription factor cocktail comprising ASCL1, BRN2, and MYT1L was identified to be the most efficient for transdifferentiating mouse embryonic and postnatal fibroblasts into induced neurons (Vierbuchen et al., 2010). This combination was identified from a pool of 19 transcription factors implicated in neuronal development. ASCL1, BRN2, and MYT1L in conjunction with NEUROD1 were also able to transdifferentiate human fetal and adult fibroblasts into induced neurons (Pang et al., 2011). Further efforts with different transcription factor combinations were able to generate specific types of neurons. For example, a combination of FOXG1, SOX2, ASCL1, DLX5, and LHX6 transdifferentiated mouse fibroblasts into specific GABAergic neurons (Colasante et al., 2015). ASCL1, BRN3B, ISL1, and SOX4 converted human and mouse embryonic fibroblasts into retinal ganglion cell-like neurons (Wang et al., 2020). Additionally, small molecules and microRNAs have been used to improve the transdifferentiation efficiency of fibroblasts to neurons. For example, porcine fibroblasts were efficiently converted into induced neurons with a combination of transcription factor ASCL1 and microRNAs miR9/9* and miR124 (Habekost et al., 2020). These microRNAs repress the SWI/SNF-like BAF chromatin remodeling complex and enable induced neurons to exit the progenitor state to continue differentiating (Yoo et al., 2011).

Outside the nervous system, directed transdifferentiation has been attempted to produce cell types as part of future regenerative therapies. For example, the generation of insulin-producing pancreatic beta cells has been considered as a treatment option for patients with Type1 diabetes. The transcription factors PDX1, NEUROG3, and MAFA (PNM factors) were identified to be essential during embryonic beta-cell development (reviewed by Zhu et al., 2017). Overexpression of this cocktail through adenoviral gene delivery methods in adult somatic cells of exocrine origin, liver duct, intestine (duodenum, jejunum) and gall bladder epithelium transdifferentiated them into insulin-producing beta cell-like cells (Zhou et al., 2008; Banga et al., 2012, 2014; Hickey et al., 2013; Chen et al., 2014). Moreover, the addition of PAX4 to the PNM factor cocktail transdifferentiated human pancreatic exocrine cells into beta cell-like cells that showed potent glucose-regulating effects when transplanted into diabetic mice (Lima et al., 2016).

In the heart, cardiomyocyte regeneration is a therapeutic option to treat coronary artery disease. A screening approach using cardiomyocyte-specific promoter-driven reporter expression, FACS, and gene expression analysis showed that three transcription factors, GATA4, MEF2C, and TBX5 (GMT factors) transdifferentiated cardiac and dermal fibroblasts into induced cardiomyocytes (Ieda et al., 2010). In human fibroblasts derived from neonatal skin, fetal heart, or embryonic stem cells, the GMT factors plus ESSRG, MESP1, Myocardin, and ZFPM2 enhanced the global expression of cardiac genes and overall transdifferentiation efficiency (Fu et al., 2013).

### Mechanisms of Direct Cellular Reprogramming

Transcription factors play a multitude of genetic and epigenetic roles within cells to bring about transdifferentiation. For example, ASCL1, a proneural bHLH transcription factor is known to play the role of an “on target” pioneer factor, meaning it binds directly to all its targets and initiates gene expression by altering chromatin conformation. Alternatively, BRN2 is recruited genome-wide by ASCL1 for binding and expressing proneural genes. MYT1L on the other hand activates gene expression in open chromatin regions by enhancing the H3K27ac and H3K4me status through KMT2B, a methyltransferase (Wapinski et al., 2013; Barbagiovanni et al., 2018). In the case of pancreatic beta-cell regeneration, it was found that PDX1 initiated the pancreatic gene expression program, specification of endocrine lineage, and maturation of beta cells (Holland et al., 2002). NEUROG3 enabled cells to take up an endocrine fate by suppressing exocrine specific genes and MAFA activates insulin expression by binding to a conserved insulin enhancer element RIPE3b/C1-A2 (Matsuoka et al., 2004; Wang et al., 2010). *Mef2c* overexpression in fibroblasts initiates the switching on of genes necessary for the formation of cardiac structures, and synthesis of contractile proteins (Dodou et al., 2004). GATA4 binds to and promotes the acetylation of H3K27 loci of cardiac genes that further results in active chromatin regions especially at the enhancers for transcription (He et al., 2014). TBX5 binds to both GATA4 and MEF2C to form unique pairs that repress non-cardiac genes in both developing and induced cardiomyocytes (Steimle and Moskowitz, 2016).

DNA methylation also plays a role in silencing non-specific gene memory signatures during direct reprogramming. During fibroblast reprogramming into induced neurons, accumulation of mCH and mCG hypermethylation marks serves a repressive function to silence fibroblast and myogenic fates (Luo et al., 2019). In sensory organs such as the retina, DNA methyltransferases (*Dnmt1, Dnmt3a, Dnmt3b*) are expressed in abundance during embryonic ages and co-operate during the formation of photoreceptors and retinal neurons in the mammalian eye (Singh et al., 2017). The expression pattern of DNMTs at postnatal ages reveals their role in the differential remodeling of cell types such as cones and rods (Nasonkin et al., 2011). Conditional knockdown of *Dnmt1* led to the aberrant apicobasal polarity of retinal pigment epithelium and neural retina differentiation (Nasonkin et al., 2013). The DNA methylation status of developing embryonic and post-natal cochlear sensory epithelia of mice has been established through whole-genome bisulfite sequencing (Yizhar-Barnea et al., 2018). In a rat aging model, hypermethylation of Connexin 26 promoter regions resulted in low expression levels and concomitant age-related hearing loss (Wu et al., 2014). Currently, there is no data on DNA methylation studies concerning hair cell reprogramming in the inner ear.

### Mechanisms of Direct Cellular Reprogramming by Pioneer Factors

A comparison of individual transcription factors highlight the fact that a select group, namely pioneer factors, has a significantly higher reprogramming ability. Pioneer factors are unique in their interactions with unmarked (no histone modifications), silent chromatin to induce transcription of genes (Zaret and Carroll, 2011; Iwafuchi-Doi and Zaret, 2014, 2016). They do this by recruiting other cofactors (activators or repressors) that by themselves are unable to interact with the silent chromatin (Gualdi et al., 1996; Carroll et al., 2005; Sekiya and Zaret, 2007). The transcription factors OCT4, SOX2, KLF-4, three of the four Yamanaka factors for pluripotency are known pioneer factors (Soufi et al., 2012, 2015). Similarly, ASCL1 in neuronal reprogramming, FOXA2 resulting from Neurogenin-3 regulation during pancreatic beta-cell reprogramming, and GATA4 in cardiac reprogramming are all pioneer factors (Bossard and Zaret, 1998; Ejarque et al., 2013; Wapinski et al., 2013). This suggests that many successful reprogramming factor combinations require pioneer factor activity for efficiently driving and establishing cell fate changes in a target cell type (Morris, 2016). Fine-tuning overexpression strategies while introducing these factors into target cells need to be explored thoroughly for obtaining completely reprogrammed cells.

### Selection and Optimization of Transcription Factors for Direct Cellular Reprogramming

A complete understanding of the reprogramming potential of the ~2,000 currently identified transcription factors by testing them individually and in combinations on approximately 250 different cell types would be an arduous trial and error-based experimental ordeal. The development of meticulous computational approaches involving several algorithms, databases, experimental results, and prediction programs (summarized in Table 1) have helped identify many “reprogramming factor/s—cell type” pairs for subsequent *in vitro* and *in vivo* testing. Transdifferentiation of multiple cell types like neurons, immune cells, pancreatic beta cells, cardiac muscle cells, and fibroblasts have been promoted for addressing cardiac and neurodegenerative diseases (Graf and Enver, 2009; Vierbuchen et al., 2010; Ladewig et al., 2013; Morris and Daley, 2013; Morris, 2016).

**Table 1:** Computational approaches developed to predict transcription factor/s (TF) suitable for reprogramming one somatic cell type to another.

No.	Model type	Approach incorporated	Validation status	Reference
1.	Expression reversal based	Data-driven approach. Representation and analysis of gene expression data as gene pairs. Identification of each gene’s strength in cell type reversal based on calculated normalizations.	No new experimental validation available	Heinäniemi et al. (2013)
2.	Polycomb repression TF model	A data-driven approach using ChIP seq and RNA seq data. The model predicts that all those TFs strongly polycomb repressed in the source cell and highly expressed in target cells are reprogramming factors for that cell pair.	No new experimental validation available	Davis and Eddy (2013)
3.	TF Cross repression model	The model predicts the reprogramming effect of unique gene set perturbations based on their influence on the stability of cell fate-specific gene networks. No prior knowledge of candidate genes/pathways was considered.	No new experimental validation available	Crespo and del Sol (2013)
4.	Epigenetic landscape mathematical model	Employing 63 cell fates and 1337 TFs from mouse microarray gene expression data, a predictive epigenetic model was built to identify hybrid cell fates, known reprogramming factors, new factors that could reprogram specific cell types.	No new experimental validation available.	Lang et al. (2014)
5.	CellNet	Gene regulatory network-based approach to compare engineered cells to target cells. New reprogramming factors were identified to uncover transitionary cellular programs and enhance the quality of engineered cells to mimic target cells.	CellNet results were tested on the conversion of B cells into macrophages. A new intestinal program was identified and fine-tuned in mouse fibroblasts reprogrammed to hepatic cells.	Morris et al. (2014)
6.	Candidate core TF atlas	An entropy-based method used to identify and build an atlas of candidate core TFs across a range of human cell types.	Results obtained from this model were tested on the conversion of human fibroblasts into induced retinal pigment epithelial-like cells.	D’Alessio et al. (2015)
7.	Mogrify	Integration of gene expression data and regulatory network information to predict reprogramming factors. A method applicable to diverse sets of TFs and cell types.	Results tested on the induction of keratinocytes from dermal fibroblasts, induction of microvascular endothelial cells from keratinocytes.	Rackham et al. (2016) and Ouyang et al. (2019)
8.	Stem cell differentiation model	Exclusive stem cell differentiation factor prediction model based on gene regulatory networks.	Results tested on neural stem cells. Overexpression of RUNX2 and ESR1 reprogrammed neural stem cells to neuronal and astrocyte cell fate, respectively.	Okawa et al. (2016)

The above examples shed light on the therapeutic applications of reprogramming factor overexpression. Despite all the promising data on reprogramming, there are certain recurrent themes on its limitations that need to be addressed. First, in almost all cases of reprogramming the resultant cells are found to be immature at several levels (all studies summarized above mention this aspect as a caveat). A specific example is that in macrophages obtained from reprogrammed fibroblasts, there is residual fibroblast gene expression, instability, and de-differentiation once the reprogramming factor expression ceases (Feng et al., 2008). Thorough reasoning and analysis into why this may be the case has shed some light on the fact that target cells may pass through a series of intermediate phases during reprogramming (pluripotent, multipotent, and precursor; Bar-Nur et al., 2015; Maza et al., 2015; Morris, 2016). These observations suggest the fidelity of reprogramming factors in truly “direct” cell fate conversions may be improbable, inefficient, and may require the transient acquisition of progenitor or stem cell states for efficient conversion.

### Considerations of Direct Cellular Reprogramming in the Inner Ear

In addition to the above considerations that have been discovered during reprogramming studies, the inner ear poses several challenges concerning reprogramming outcomes and their success rates. Employing reprogramming factors to convert iPSCs or fibroblasts *in vitro* into hair cells that may be eventually transplanted, is possible in many tissues where the cellular organization is not paramount, but unlikely to succeed in the inner ear where the precise number and location of sensory cells is crucial to their function, and the mechanical properties of the cochlea. Alternatively, the reprogramming of abundantly available cells that serve as a reservoir within the inner ear tissue, such as supporting cells of the organ of Corti or the adjacent non-sensory cells of the inner and outer sulci may also be attempted. Many non-sensory cell reservoirs exist within the mammalian inner ear and show evidence for their responsiveness to transcription factor-mediated reprogramming into induced hair cells. We discuss these transcription factors and the cell types capable of being reprogrammed in further sections.

## Transcriptional Control of Hair Cell Regeneration in Non-Mammalian Vertebrates

Non-mammalian vertebrates such as fish, birds, and amphibians are known to regenerate hair cells in response to noise or chemical damage, as well as replacing hair cells through physiological turnover under normal, undamaged conditions (Cotanche, 1987; Cruz et al., 1987; Corwin and Cotanche, 1988; Ryals and Rubel, 1988; Lippe et al., 1991; Lombarte et al., 1993; Taylor and Forge, 2005; Smith et al., 2006). This regeneration process can occur by asymmetric division of supporting cells to give rise to hair cells, as well as direct transdifferentiation of supporting cells into hair cells in the absence of cell division (Adler and Raphael, 1996; Roberson et al., 1996; Baird et al., 2000). Zebrafish and chicken are useful non-mammalian vertebrate models to study the inner ear, owing to the conservation of inner ear development genes between these species and mammals (Gates et al., 1999; Barbazuk et al., 2000; Chan et al., 2009). Additionally, delineating the molecular and genetic differences between a regenerating and non-regenerating system may shed light on potential strategies to regenerate hair cells in mammals.

The proneural family of transcription factor genes was found to be important for the generation of neurons, and also cells that differentiated into sensory organs (Ghysen and Dambly-Chaudiere, 1989; Bertrand et al., 2002). bHLH transcription factors include the proneural genes *atoh1*, *neurog1–3*, and *neurod1* (Murre et al., 1989). In zebrafish, the *atoh1* homologs *atoh1a* and *atoh1b* are required for hair cell development (Millimaki et al., 2007). Expression of *atoh1a* in support cells along with disruption of Notch signaling gave rise to supernumerary hair cells that eventually did not survive (Itoh and Chitnis, 2001; Itoh et al., 2003). In addition to *atoh1a*, another proneural factor, *neurod*, was found to be expressed in the zebrafish lateral line. Loss of function of either *atoh1a* or *neurod* resulted in the loss of hair cells (Sarrazin et al., 2006). In the chicken inner ear, ATOH1 is involved in hair cell development and regeneration, just as in zebrafish. Hair cells require sensory lineage specification by SOX2 and subsequent differentiation driven by ATOH1 (Neves et al., 2012). Regeneration of hair cells in the basilar papilla of birds occurs only in the event of hair cell death or damage and occurs by supporting cells transdifferentiating into hair cells, either directly or after re-entering the cell cycle (Tsue et al., 1994). This process is mediated by upregulation of ATOH1 in supporting cells (Cafaro et al., 2007)—for example, 15% of supporting cells labeled by a BrdU pulse given 4 days after deafening expressed ATOH1 within 2 h of the pulse. These early studies confirmed the conservation and importance of ATOH1 homologs in the development and regeneration of vertebrate auditory hair cells.

Independent studies in zebrafish and chicken identified additional factors important for hair cell regeneration. For example, *sox2*, a known pluripotency transcription factor, was shown to be involved in the regulation of hair cell regeneration in zebrafish (Millimaki et al., 2010). Overexpression of a combination of *sox2* and *atoh1a* resulted in an enhanced number of ectopic hair cells in the zebrafish lateral line compared to either one alone (Sweet et al., 2011). A bulk RNA-seq analysis performed on support cells and mantle cells (a stem cell population in the zebrafish lateral line) showed that the Notch and Fgf signaling pathways were significantly downregulated in early hair cell regeneration (Jiang et al., 2014). In chicken, a large-scale gene expression analysis study focusing on identifying differentially expressed genes in regenerating utricles identified 15 transcription factors whose expression correlated with regeneration (Ku et al., 2014). These included functionally unique genes like BTG1 that appear to promote hair cell differentiation but negatively regulate proliferation (Rouault et al., 1992; Rodier et al., 1999), and factors that had not previously been associated with regeneration such as IRF-1 and CITED4. Among highly expressed transcription factors were targets of the Notch signaling pathway, including MAMLD1, RBPJ, the ID family genes (ID1, ID4, ID2), ATOH1, and HEYL (Ku et al., 2014).

## Hair Cell Regeneration in Mammals

The cochlea of neonatal mammals possesses a limited capacity for spontaneous hair cell regeneration in response to hair cell death. Newborn mouse supporting cells respond to signals from dying hair cells and regenerate new hair cells through mitotic division or direct transdifferentiation (Cox et al., 2014). Mechanistic studies have shown Wnt, Notch, and ERBB2 signaling pathways to be essential for spontaneous hair cell regeneration in neonatal mice (Hu et al., 2016; Ni et al., 2016; Zhang et al., 2018). Specific manipulations involving these signaling pathways have been explored for mammalian hair cell regeneration. For example, Wnt pathway activation or Beta-catenin overexpression led to the proliferation of hair cell progenitors that differentiated eventually into hair cells (Chai et al., 2012; Shi et al., 2014). Inhibition of the Notch pathway is known to upregulate ATOH1 in neonatal supporting cells, enabling their transdifferentiation into hair cells (Korrapati et al., 2013; Mizutari et al., 2013). Although such attempts were successful in regenerating hair cells in young, pre-hearing animals, their ability to achieve similar results in older animals failed. To regenerate hair cells in older animals, the overexpression of transcription factors to reprogram nonsensory inner ear cells into hair cells is a promising approach. We discuss a variety of non-sensory cell types that are potential targets for reprogramming in the mammalian cochlea.

### Inner Ear Non-sensory Cells: Potential Targets for Transcription Factor Reprogramming

**Figure 1:**
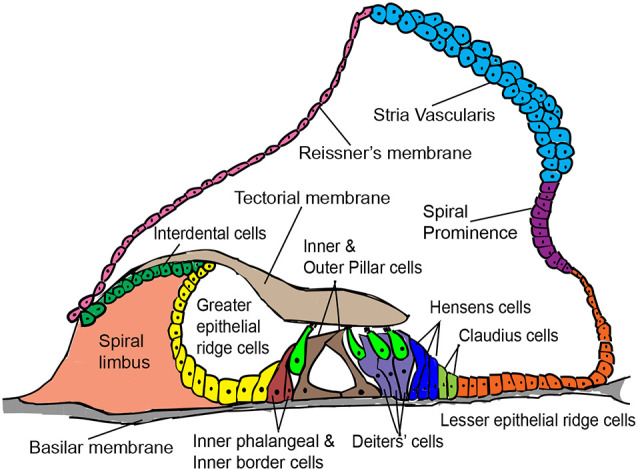
Schematic cross-sectional view of the postnatal mammalian organ of Corti, denoting some structural features and a variety of cell types of interest for *in vivo* hair cell reprogramming.

The various non-sensory cell types of the mammalian cochlea are indicated in Figure 1. Supporting cells lie adjacent to hair cells in the organ of Corti and are the most suitable for hair cell regeneration through transcription factor reprogramming. Developmentally, supporting cells and hair cells arise from common progenitors in the sensory patch of the cochlea. The differentiation and patterning of the two cell types are influenced by the expression of several genes and signaling pathway members, such as Notch signaling (reviewed by Basch et al., 2016). Supporting cells are broadly classified into inner border cells, inner phalangeal cells, pillar cells (inner and outer), Hensen cells, Deiters’ cells, and Claudius cells (Raphael and Altschuler, 2003). Previous studies have shown that supporting cell-specific damage results in the regeneration of inner border and phalangeal cells but not pillar or Deiters’ cells in neonatal mice (Mellado Lagarde et al., 2013, 2014).

Greater epithelial ridge (GER) cells are columnar cells lying adjacent to the inner hair cell layer of the organ of Corti. They are a transient population of cells occurring in neonatal animals and undergo thyroid hormone-dependent remodeling between 1 and 2 weeks of age (Sharlin et al., 2011; Peeters et al., 2015). This remodeling involving programmed cell death and cell shape changes, creates the inner sulcus, a cavity filled with short cuboidal epithelial cells that promote free movement of hair cell stereocilia against the tectorial membrane during sound transduction (Hinojosa, 1977; Kamiya et al., 2001). The physiological plasticity of GER cells that enable their remodeling into the inner sulcus has also been exploited to regenerate hair cells through the ectopic expression of *Atoh1* in the neonatal mouse cochlea (Kelly et al., 2012), which we discuss further below.

Interdental cells are present medial to the GER region and are the point of attachment for the tectorial membrane. The interdental cells secrete components of the tectorial membrane matrix and are involved in K^+^ recycling for hair cell function (Lim, 1972; Spicer et al., 1999). Lesser epithelial ridge (LER) cells lie adjacent to the outer hair cells, comprise the Hensen’s and Claudius supporting cell types, are lateral to the organ of Corti, and eventually form the outer sulcus region. When *Atoh1* is induced in cochlear LER cells *in vitro*, they differentiate into hair cell-like cells (Zhai et al., 2005). Interestingly, the GER, LER, and interdental cells arise from the same pool of *Eya1^+^* multipotent progenitors that give rise to hair cells and supporting cells during inner ear development (Xu et al., 2017). Currently, there are no *in vivo* reprogramming attempts targeting the LER and interdental cells for hair cell regeneration.

### ATOH1–Inner Ear Development, Context-Dependence, and Reprogramming

The mammalian inner ear is derived from an ectodermal thickening named the otic placode, developing on either side of the embryonic hindbrain. The transient expression of the *Sox2* transcription factor in a specific population of cells in the cochlear duct marks the prosensory domain followed by the expression of *p27kip1* that drives sensory cells to become post-mitotic (Lee, 2006). The ATOH1 transcription factor is then expressed in a cluster of prosensory cells and a hair cell differentiation program is initiated (Bermingham et al., 1999; Woods et al., 2004; Driver et al., 2013). Simultaneously, the expression of Notch ligands is induced in these nascent hair cells, thereby inhibiting a hair cell fate in adjacent cells through lateral inhibition (Lanford et al., 1999; Kiernan et al., 2005). The *Atoh1* promoter regions in these adjacent cells undergo rapid repression through induction of Hes/Hey genes, causing them to adopt a supporting cell fate (Abdolazimi et al., 2016). Three-dimensional live imaging of ATOH1 activity in cochlear explants showed that hair cell induction occurs with the formation of inner hair cells (medial) followed by that of outer hair cells (lateral) (Tateya et al., 2019). In mammals, hair cell differentiation starts near the base and ends at the apex of the cochlea. After this initial developmental phase, ATOH1 is also necessary for the survival and proper function of hair cells (Pan et al., 2012; Cai et al., 2013; Chonko et al., 2013).

ATOH1 plays the most important role in hair cell fate specification, implicating it as a master regulator (a single factor determining a unique cell fate). ATOH1 independently recognizes and binds to specific E-box motifs in the promoter and enhancer regions of its targets (Powell et al., 2008). However, the set of targets it regulates is variable between ATOH1-expressing cell types, implying that ATOH1 acts in a context-dependent fashion to promote cellular differentiation. Transcriptomic characterization of neonatal hair cells has identified hair cell-specific ATOH1 target genes of which a small number overlap with those found in ChIP-seq data from the cerebellum and intestine (Cai et al., 2015). Several mechanisms may promote the specificity of ATOH1’s targets in hair cells. First, the transient expression of *Sox2*, a pioneer factor ahead of *Atoh1* which is unique to hair cell differentiation, results in a changing chromatin landscape enhancing chromatin accessibility for ATOH1 to bind to its targets in hair cell progenitors (Kempfle et al., 2016). Second, differential control of ATOH1 activity through the phosphorylation of a serine moiety in its bHLH domain which acts as a switch to control ATOH1’s DNA binding ability in a variable manner across tissues (Quan et al., 2016; Xie et al., 2017). Third, within the inner ear hair cells, ATOH1’s expression is tightly temporally regulated by a series of histone modifications of its promoter and enhancer regions H3K4me3/H3K27me3, H3K9ac and H3K9me3. These marks enable ATOH1 to rapidly and dynamically transition from a poised to an active state during hair cell specification, and to render the ATOH1 locus in a repressive state postnatally in supporting cells (Stojanova et al., 2015).

The importance of *Atoh1*’s expression in hair cell development made it an excellent candidate for reprogramming to promote hair cell regeneration. *Atoh1* overexpression in postnatal cochlear and utricle explants from rat inner ears transdifferentiated nonsensory cells into ectopic hair cells (Zheng and Gao, 2000; Shou et al., 2003). Mouse embryonic stem cells transdifferentiated *in vitro* into hair cell-like cells (expressing cochlear hair cell markers) in response to ectopic expression of *Atoh1* (Ouji et al., 2013). A transcriptomic study showed that induced multipotent otic progenitors showed a profound “pro hair cell” effect compared to mouse embryonic stem cells in response to *Atoh1* overexpression (Ebeid et al., 2017). Early *in vivo* ATOH1 gene therapy studies employing adenoviral gene delivery methods in normal and deafened adult guinea pig cochleae showed regeneration of hair cells and improvement of hearing thresholds (Kawamoto et al., 2003; Izumikawa et al., 2005). However, these studies also highlight several confounding aspects, such as tissue damage in response to viral inoculation into the endolymph. Initial *in vivo* studies in the neonatal mouse cochlea, which investigated the effect of ATOH1 on reprogramming non-sensory cells into hair cells, employed transgenic mice harboring an inducible *Atoh1* transgene. Histological analysis post-overexpression showed that the greater epithelial ridge cells, as well as a small percentage of pillar and Deiters’ cells, could be reprogrammed to hair cells (Kelly et al., 2012; Liu et al., 2012). Among supporting cells, the efficiency of ATOH1 reprogramming was much better when targeted to the inner phalangeal and border cells in the neonatal mouse cochlea, but virtually non-existent in Deiters’ or pillar cells. The ectopic hair cells expressed several hair cell-specific markers and showed minimal synaptic density (Liu et al., 2014). However, the competence of these cells to become hair cells in response to ATOH1 alone declined rapidly with age, challenging the feasibility of employing this strategy for hair cell regeneration in older animals (Kelly et al., 2012; Liu et al., 2012). This prompted the search for other transcription factors in addition to ATOH1 that could enhance reprogramming efficiency for auditory hair cell regeneration, by analogy to the direct reprogramming studies in other tissues that we described earlier in the review.

### Hair Cell Reprogramming Strategies Employing ATOH1 in Combination With Other Reprogramming Partners

#### ATOH1 and POU4F3

The POU-IV domain transcription factor, POU4F3 (also BRN-3C) is a downstream target of ATOH1 and is induced after the onset of *Atoh1* expression in inner ear hair cells [validated computationally and through ChIP experiments by Masuda et al. (2011, 2012)]. ATOH1 regulates *Pou4f3* expression synergistically with GATA3, MYC, and TFE2 (Ikeda et al., 2015). POU4F3 plays a major role in the maturation and survival of hair cells (Xiang et al., 1998). Deletion of *Pou4f3* in the mouse inner ear leads to severe morphological deficits and apoptosis of hair cells (Xiang et al., 1997, 1998). A combination of ATOH1, POU4F3, and GATA3 was able to reprogram mature supporting cells into hair cell-like cells in the adult cochlea. This study also provided evidence for the *p27kip1* gene playing a critical role in preventing ATOH1 mediated transdifferentiation of supporting cells by down-regulating GATA3 in mature cochlear supporting cells (Walters et al., 2017), although whether the P27 protein is functioning in this context as a cyclin-dependent kinase inhibitor or mediating an additional function is not clear.

#### ATOH1 and GFI1

GFI1 (Growth factor independent 1) is a zinc-finger transcription factor expressed in hair cells and cochlear neurons during development (Wallis et al., 2003). Loss of function studies in mice has shown that GFI1 does not disrupt hair cell specification but affects later-stage morphology and survival of hair cells. In *Gfi1* null mice, outer hair cells followed by inner hair cells undergo apoptosis that is complete by 2 weeks of age, although vestibular hair cells survive, albeit in an abnormal condition (Wallis et al., 2003; Hertzano et al., 2004). An *in vivo* translatome analysis performed using *Gfi1Cre;RiboTag* mice showed that in the absence of GFI1, neuronal fate genes such as *Pou4f1* were upregulated in hair cells (Matern et al., 2020). Hence, GFI1 may play a dual role in fine-tuning hair cell differentiation by repressing non-hair cell genes (particularly neuronal genes), in addition to enabling the expression of hair cell-specific genes. *In vivo* studies employing a hair cell damage model (*Pou4f3DTR*) in adult mice showed that adenoviral delivery of ATOH1 and GFI1 together post hair cell ablation in the organ of Corti led to the transdifferentiation of supporting cells to give rise to hair cells at a significantly higher efficiency than ATOH1 alone (Lee S. et al., 2020).

#### ATOH1 and ISL1

Islet-1 (ISL1) is a LIM domain transcription factor and like ATOH1, ISL1 behaves in a context-dependent manner in neuronal and non-neuronal tissue types (Hobert and Westphal, 2000). It is an early marker of both the prosensory domain and spiral ganglion neuron in the developing otic placode (Radde-Gallwitz et al., 2004; Huang et al., 2013), but is also expressed transiently in hair cells (Cai et al., 2015). Early overexpression of *Isl1* in the inner ear results in an age-related hearing loss phenotype in mice (Chumak et al., 2016). However, overexpression of *Isl1* in postnatal mouse cochlear hair cells specifically protects them from damage due to age or noise with no functional anomaly (Huang et al., 2013). Ectopic co-expression of both *Atoh1* and *Isl1* in neonatal cochlear explants *in vitro* and neonatal mice *in vivo* resulted in a significantly higher number of reprogrammed hair cells as compared to overexpression of *Atoh1* alone (Yamashita et al., 2018).

#### ATOH1, GFI1, and POU4F3

*In vitro* studies in mouse embryonic stem cells and chick otic epithelial cells showed that overexpressing *Atoh1* alone drove them to adopt a neuronal fate (Costa et al., 2015). In contrast, a combination of ATOH1, POU4F3, and GFI1 induced many hair cell genes when misexpressed in mouse embryonic stem cells. These induced hair cells expressed characteristic markers, possessed hair bundle-like projections and their transcriptome indicated elements of a hair cell signature (Costa et al., 2015). The GAP factors together with another transcription factor, SIX1, reprogrammed mouse embryonic fibroblasts and adult tail-tip fibroblasts *in vitro* into induced hair cells. In addition to what was seen in the previous study, these induced hair cells possessed a hair cell-like epigenetic profile, electrophysiological properties, expression of transduction channel proteins, and sensitivity to ototoxins (Menendez et al., 2020).

#### Thoughts on Additional Reprogramming Factors—SOX2, GATA3, EYA1, and SIX1

In addition to the above reprogramming factor combinations, SOX2, GATA3, EYA1, and SIX1 are additional reprogramming factor candidates whose combinatorial overexpression shows promise to induce hair cells, based on their expression pattern and co-operativity. For example, analysis of open chromatin regions of prosensory cells indicated that binding sites for SOX2, GATA3, and SIX1 were highly enriched, implying these genes play a critical downstream role for hair cell differentiation (Wilkerson et al., 2019). The transient expression of SOX2 preceding ATOH1’s hair cell specification role is governed by the activity of SIX1 that in turn downregulates SOX2 (Zhang et al., 2017). SIX1 was found to be a hair cell selector gene that governs the sequence of events for hair cell differentiation. It does so by occupying enhancer regions of its target genes which are transcribed, and physically interacting and synergizing with the GFI1, ATOH1, and POU4F3 factors and GATA3 (Li et al., 2020). The interaction of EYA1 and SIX1 is necessary in addition to SOX2 expression for the induction of ATOH1 in the developing mouse cochlea (Ahmed et al., 2012). *In vivo* analysis of GATA3’s role in the prosensory domain indicate its involvement in *Atoh1* upregulation and spiral ganglion neuron development (Duncan and Fritzsch, 2013). These factors have the potential to supplement the GFI1/ATOH1/POU4F3 factors and thereby enable fine-tuning of hair cell reprogramming efficiency.

## Conclusion and Future Perspectives

**Figure 2:**
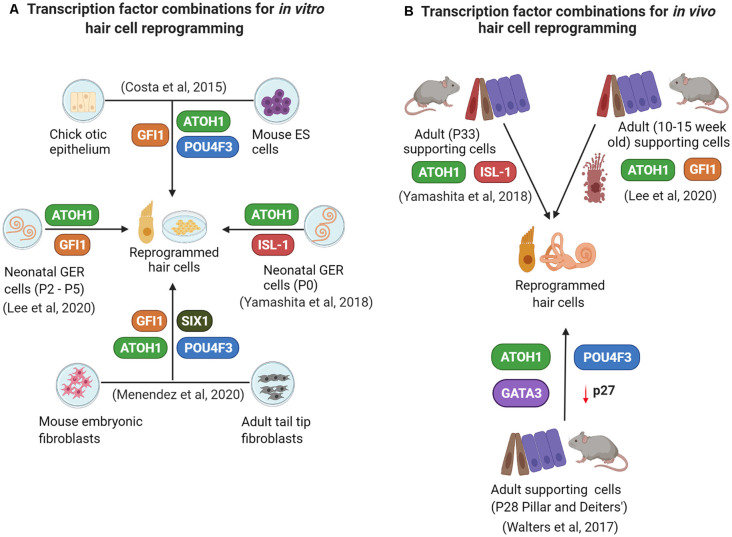
A summary of some current *in vitro* **(A)** and *in vivo* **(B)** reprogramming studies employing overexpression of different transcription factor combinations.

Successful reprogramming of non-sensory cells into hair cells (summarized in Figure 2) in the mammalian inner ear is a promising approach to restore auditory function. Reprogramming factors that have been shown to drive cells towards a hair cell fate when overexpressed together include ATOH1, GFI1, POU4F3, and SIX1. Selective inclusion of other factors and perturbations of critical pathways like Notch, Wnt, and Fgf signaling may be necessary, in addition to epigenetic remodeling of the target cell population to make their chromatin more accessible to reprogramming factors. There is a good chance that an optimal reprogramming factor code may differ slightly between different “starter” cell types that have to be reprogrammed into hair cells. Supporting cells remain the target cells of choice for reprogramming based on their proximity to hair cells. However, just as in non-mammalian vertebrates, replacement of the reprogrammed supporting cells will be necessary to preserve normal cochlear mechanics. We discuss these, some additional questions, and the challenges of hair cell reprogramming below.

### To What Extent Are Reprogrammed Hair Cells Functional, and Can They Restore Function in the Damaged Auditory or Vestibular System?

Most reprogramming studies to date have evaluated the reprogramming outcome primarily from a genetic and protein expression perspective with less focus on hair cells, and overall auditory or vestibular function. Testing the mechanotransduction ability of reprogrammed hair cells through electrophysiological studies is an important assay for individual reprogrammed hair cell function. Moreover, it is also essential to evaluate the higher-order functional consequences of reprogramming through audiological and/or vestibular testing. In this regard, it is notable that regenerative reprogramming of vestibular hair cells in the mouse utricle has recently been shown to restore aspects of vestibular function over several months (Sayyid et al., 2019).

### For How Long Do Reprogrammed Cells Survive, and Are They a Long-Term Solution to Hearing or Balance Defects?

The survival and maturation of reprogrammed hair cells are necessary for long-term auditory function. From prior studies and our unpublished data, we know that reprogrammed hair cells derived from supporting cells and GER cells do not survive for more than a few weeks *in vivo* in the mammalian cochlea. Even within this time frame, reprogrammed hair cells lack certain intricate developmental features like planar cell polarity and frequency tuning properties, as seen by the haphazard, non-directional stereocilia arrangement, which could be due to defects in individual cells, or disorganization caused by excess hair cell production (Kelly et al., 2012). Future reprogramming studies need to focus on maximizing the extent of hair cell maturation and survival to aim for long-term function. A striking example of the consequences of suboptimal hair cell differentiation on survival was recently observed in mice carrying a single point mutation of *Atoh1*. The Atoh1S193A variant in the bHLH domain does not appear to affect transcription in reporter assays, yet this mutation causes progressive cochlear hair cell degeneration (Xie et al., 2017).

### Can Reprogramming Generate Hair Cell Subtypes?

Hair cells can be divided broadly into inner and outer hair cells of the auditory system, and type I and II hair cells in the vestibular system. Moreover, regional differences are known to exist in a given inner ear sensory organ, such as the significant differences in cell and hair bundle size along the tonotopic axis of the cochlea. At present, it is not clear when each type of hair cell is specified, what signals are responsible for subtype specification, and whether current reprogramming cocktails favor one hair cell subtype over another, as has been seen with current protocols that generate largely vestibular hair cells in embryonic stem cell- or iPS cell-derived organoids (Longworth-Mills et al., 2016; Koehler et al., 2017). There may be a need for other reprogramming factors that will play a “subtype specification” role. For example, INSM1 is a zinc finger transcription factor family member unique in expression to outer hair cells of the mammalian cochlea (Lorenzen et al., 2015). *Insm1* deletion in the neonatal cochlea leads to the expression of inner hair cell-specific genes in outer hair cells (Wiwatpanit et al., 2018). Similarly, IKZF2/Helios is another outer hair cell transcription factor whose overexpression upregulates outer hair cell-specific genes and confers electromotility characteristics to target cells (Chessum et al., 2018). In the vestibular system, EMX2 is a transcription factor known for its role in controlling hair bundle orientation across the line of polarity reversal in the mouse utricle (Jiang et al., 2017).

### Will Prolonged Overexpression of Reprogramming Factors Pose Long-Term Challenges Post Hair Cell Reprogramming?

During the development of the mouse organ of Corti, the expression of some hair cell transcription factors such as ATOH1 are transient, and some are present in progenitor cells before restricting to hair cells. Current *in vivo* hair cell reprogramming strategies generally drive constant expression of reprogramming factors, and so it is possible that the persistence of factors whose expression is normally downregulated in hair cells may compromise their mature function or may hold the reprogrammed hair cells in a permanently immature state. More studies are required to determine whether this will impede regeneration driven by reprogramming. Recent advances in the delivery of encapsulated RNA or DNA editing molecules or fusion of proteins to cell-penetrating peptides may offer a way to transiently deliver reprogramming factors to the ear (Takeda et al., 2016; Gao et al., 2018).

### What Are the Consequences of Losing Reprogrammed Cells as They Convert Into Hair Cells?

Inner border and inner phalangeal cells lie adjacent to inner hair cells and exhibit higher plasticity towards reprogramming as opposed to Hensen cells, Deiters’ cells, and Claudius cells that lie adjacent to outer hair cells (Liu et al., 2014; Figure 1). The question of why some supporting cells are harder to reprogram as compared to others, even in early postnatal ages remains unanswered. Nevertheless, supporting cell reprogramming is the most optimal regeneration strategy given their physical proximity to hair cells. However, the loss of supporting cells by transdifferentiation without supporting cell division will pose a challenge for the hearing function, as studies have shown that supporting cell loss disrupts auditory function (reviewed by Wan et al., 2013). Similarly, the timely remodeling of GER cells into the inner sulcus is another critical event that ensures correct auditory function, and any change in GER remodeling may affect hearing directly (Peeters et al., 2015). So, evaluating the long-term loss of these cell types and/or considerations to regenerate these cells is essential.

### How Efficient Is the Reprogramming Process From the Perspective of Upregulated Hair Cell Gene Regulatory Networks and Silenced Target Cell Gene Networks?

Detailed gene expression and gene regulatory network analyses are necessary to fully understand the reprogramming efficiency of a transcription factor cocktail as a target cell transitions from its original fate to a specific final fate. For example, single-cell mapping studies for delineating cell reprogramming identity and lineage (Biddy et al., 2018). However, the complexity of the reprogramming process and the fact that it is influenced by variables such as age and epigenetic state make this testing more challenging in comparison to the functional studies required above. Epigenetic landscapes of both the target cell and the reprogrammed cell are controlled by specific transcription factor combinations used in reprogramming. As mentioned earlier, cell types respond more completely to reprogramming when a pioneer factor is overexpressed along with other transcription factors (Morris, 2016). A detailed understanding of how pioneer factors like POU4F3 and SOX2 alter the epigenetic landscapes of the target cell towards that of hair cells can be determined through ATAC sequencing experiments. With the advent of newer high-throughput technologies and bioinformatics pipelines, addressing reprogramming efficiency will become more tractable in the future, leading to better strategies to drive reprogramming and regeneration in the inner ear.

### What is the Best *In vivo* Gene Delivery Strategy for Hair Cell Reprogramming Employing Transcription Factors?

The application of reprogramming factors as a therapeutic strategy for hair cell regeneration requires optimal gene delivery *in vivo*. Like the retina, the inner ear is an attractive tissue for targeted therapies as it is relatively well-enclosed and isolated from the central nervous system and circulatory system. For inner ear-specific therapies, various anatomical routes have been tested, including approaches through the posterior semicircular canal, round window, and cochleostomy (reviewed by Ahmed et al., 2017). Non-viral methods of gene delivery through cationic lipid nanoparticles (to deliver Cas9 guide RNA lipid complexes specific for *Tmc1* allele) have been successful in reducing hearing loss in mice (Gao et al., 2018). Other modes of delivering genes, proteins, drugs, and siRNAs molecules include hydrogel encapsulation, exosomes, PLGA nanoparticles, and supra-particles (reviewed in Ma et al., 2019). The limitation of these unique delivery systems is that not all regions of the inner ear can be accessed and that restricts the scope of its application for treatment. However, the transient nature of these treatments may reduce the potential negative consequences of over-expressing transcription factors for extended periods.

Gene delivery through viral methods includes the use of adenovirus, adeno-associated-virus, lentivirus, and exosome-associated adenoviruses. Studies in animal models have shown that adeno-associated viruses are effective in targeting hair cells for hearing loss gene therapy (Landegger et al., 2017; Akil et al., 2019; Isgrig et al., 2019; Nist-Lund et al., 2019). AAVs were found to have low overall toxicity due to minimal immune response to it by the host and low rate of host genome integration (Nakai et al., 2001). Genetically engineered and improvised AAV9-PHP.B was found to be efficient for targeting hair cells in the organ of Corti in mice and primates (György et al., 2019; Lee J. et al., 2020). Though not tested directly, similar technologies will likely be effective in targeting non-sensory and supporting cells for hair cell reprogramming.

## Author Contributions

Both authors wrote and edited the manuscript. AI prepared the figures. All authors contributed to the article and approved the submitted version.

## Conflict of Interest

The authors declare that the research was conducted in the absence of any commercial or financial relationships that could be construed as a potential conflict of interest.
